# Status of metered dose inhaler technique among patients with asthma and its effect on asthma control in Northwest Ethiopia

**DOI:** 10.1186/s13104-019-4059-9

**Published:** 2019-01-14

**Authors:** Melles Mebrahtom, Nebiyu Mesfin, Hailay Gebreyesus, Mebrahtu Teweldemedhin

**Affiliations:** 1 Department of Internal Medicine, College of Medicine and Health Sciences, University of Gondar, Gondar, Ethiopia; 2 College of Health Science, Aksum University, P.O. Box 298, Aksum, Tigray Ethiopia

**Keywords:** Metered dose inhaler technique, Asthma control, Determinants

## Abstract

**Objective:**

In Asthma management, poor handling of inhalation devices and wrong inhalation technique are associated with decreased medication delivery and poor disease control. The aim of this study was to assess the status of Metered dose inhaler technique, associated factors and its impact on Asthma control among adult patients with Asthma.

**Results:**

The mean duration of Asthma was 15 ± 13 years. Asthma was uncontrolled in 70.4% of the participants and the poor technique of Asthma inhaler device was observed in 71.4% of the patients. Lack of health education on Metered dose inhaler technique [AOR =4.96; 95% CI (1.08–22.89)], and uncontrolled Asthma [AOR =3.67; 95% CI (1.85–7.23)], was independently associated with poor Metered dose inhaler technique.

**Electronic supplementary material:**

The online version of this article (10.1186/s13104-019-4059-9) contains supplementary material, which is available to authorized users.

## Introduction

Bronchial Asthma is a common, chronic inflammatory airway disease characterized by bronchial hyper reactivity with variable degree of airway obstruction [1]. Bronchial Asthma affects about 300 million people worldwide, causing 250,000 deaths per year [2]. In addition to the human burden, the economic impact of Asthma is significantly costing an estimated $20 billion every year in developing nations, the main reasons for the deaths and costs being poor disease control and treatment [3].

The goal of Asthma treatment is to achieve and maintain clinical control through a continuous cycle of assessment, treatment, and monitoring to maintain symptom control [1]. Level of Asthma control is below recommended standards in Africa and it ranges from clinically manifested Asthma, to significantly decreased or removed symptoms by treatment [4, 5].

Inhalation devices are part of the mainstay of management in Bronchial Asthma, during the long-term treatment, however, their effectiveness largely depends on the inhalation technique. Poor handling and wrong inhalation technique are associated with decreased medication delivery and poor disease control [6, 7].

There are different validated tools for assessing control of Asthma, including the global initiative for asthma (GINA) guideline which has six criteria including daytime and night-time symptoms, limitations in activities, the need for rescue medication, lung function and clinical exacerbations [8].

Another validated international tool is the Asthma control test (ACT) questionnaire, developed to facilitate and standardize the assessment of Asthma control. It is a patient-filled questionnaire with 5 items dealing with Asthma symptoms, rescue medication use, and the impact of Asthma on daily activity. ACT results has significant correlation with baseline FEV1 [9]. Poor Asthma control was associated with age at onset of Asthma, family history, smoking, obesity, low educational level, low income, co morbidity and absence of medications for Asthma [10].

In Ethiopia, studies on patients’ inhalational technique and Asthma control are scarce. Therefore, the aim of this study was to assess the status of Metered dose inhaler technique, associated factors and its impact on Asthma control among adult patients with Asthma which is vital to provide baseline information and devise evidence based solution.

## Main text

### Methods and materials

#### Study area and period

The study was conducted from May 12 to October 30, 2017 in University of Gondar hospital, one of the few university referral hospitals of Ethiopia, located in Gondar, North-West Ethiopia. The hospital gives outpatient and inpatient services for any referred and non referred cases from Gondar area. In the chest clinic where care is given for clients with Asthma, there are four Internists with inclination in pulmonology and eight nurses actively giving different services including the inhaler technique.The study period was selected considering the months with relatively high patient flow.

#### Study design and populations

A facility-based cross-sectional study design was conducted among clinically-diagnosed patients with Asthma, aged ≥ 18 years. Patients who were at chest clinic follow up during the study period were included and those who were not on inhaler medications at the time of data collection were excluded from the study.

#### Sample size and sampling techniques

The Sample size was calculated using the single population proportion formula. Because there was no previous study conducted at the nation and in the study area in this specific study group, a prevalence level that estimate maximum sample size was considered as 50%, 5% margin of error, with 95% confidence interval and 10% for non-response rate. So, the final calculated sample size was 216. Sample was taken from 400 patients who were at chest clinic follow up and then systematic random sampling technique was used to select study participants from the registration book. First, the list of patients having chest clinical appointment with their card numbers was taken from the hospital medical chronic clinic department. The interview was started by selecting a random start using lottery method.

#### Data collection tools and procedures

Primary data was collected from the study subjects using structured and pretested questionnaire. First the questionnaire was prepared in English and translated to local language (Amharic) and translated back to English to observe its consistency. Finally, the questionnaire was pre tested on 5% of patients before the actual data collection in Aksum university referral hospital correction and modification were done based on the gap identified during the interview. Two Medical residents and two Bachelors of Sciences (B.Sc.) Nurses assigned in the chest clinic were trained for 2 consecutive days they collected the data under the supervision of two senior physicians. Information pertain the socio-demographic variables was collected by the B.Sc. Nurses through direct interview of the study subjects. Additionally, the medical record of the study subjects which was previously recorded by medical doctors was used to confirm the clinical related variables. The scores of the Asthma control status and the inhalational device assessment tool (IDAT) was determined by the trained residents using the predetermined parameters and steps well controlled Asthma was defined as ACT score of ≥  20 and a score of 95% to 100% was considered for target proper techniques for IDAT (Additional file 1: Appendix S1, Additional file 2: Appendix S2).

#### Data quality control

The questionnaire was pre-tested before the actual data collection period. Formal training was given to the data collectors and supervisors and data was double checked by the immediate supervisors to avoid errors.

#### Data processing and analysis

Data was collected from the patients and the medical record charts of patients diagnosed with Asthma using structured data collection format. Data entry was made using EPI data and analyzed using SPSS software version 20. Each variable was evaluated independently in a bivariate analysis and association was determined using cross tabulation and COR (crude odds ratio) at 95% CI (confidence interval). Gender, age, religion, residence, marital status, occupation, educational status, monthly income, smoking status, co-morbidities, alcohol intake, type of MDI use, duration of MDI device use, frequency of beclomethasone, health education on MDI use, health education provider, family history of bronchial Asthma, Asthma control status and duration of Asthma were the independent variables considered in the regression analysis. All variables associated with uncontrolled Asthma at a p-value < 0.05 on the bivariate analysis was entered into a multivariate logistic regression analysis to determine variables that were independently associated with poor Metered inhaler (MDI) technique at p-value < 0.05.

### Result

#### Socio demographic characteristics of respondents

A total of 216 patients with Asthma were interviewed giving final response rate of 95.4%. The mean age of the participants was 47.41 ± 13.207 years. Females accounted for 58.1% of the study population and 63.1% of the participants were married (Table 1).

**Table 1:** Socio-demographic characteristics of patients with Asthma at University of Gondar hospital North West Ethiopia, 2017

Socio-demographic variables	Categories	Frequency (n)	Percent (%)
Sex	Male	85	41.3
	Female	121	58.7
Residence	Urban	153	74.3
	Rural	53	25.7
Marital status	Single	24	11.7
	Married	130	63.1
	Divorced	23	11.2
	Widowed	29	14.1
Age Category	18–34	35	17.0
	35–54	115	55.8
	≥ 55	56	27.2
Religion	Orthodox	162	78.6
	Muslim	29	14.1
	Protestant	15	7.3
Occupational	Merchant	24	11.7
	Civil Servant	55	26.7
	House Wife	76	36.9
	Student	51	24.8
	Merchant	24	11.7
Educational status	No formal education	112	54.4
	Primary school	50	24.3
	Secondary school	38	18.4
	Higher education	6	2.9
Family income	≤ 1200	87	42.2
	1201–2499	106	51.5
	≥ 2500	13	6.3

#### Clinical related variables of respondents

The mean duration of Asthma was 15 ± 13 years and Asthma was uncontrolled in 70.4% of the participants (Table 2). With regards to the type of inhalational drugs, the majority were using both beclomethasone and salbutamol (72.3%) and 85% of the respondents were taking beclomethasone in twice daily base. Although majority of the participants had improper MDI technique (71.4%), about 86.9% responded that they have received health education on MDI technique (Table 2).

**Table 2:** The frequency of clinical related variables among patients with Asthma (n = 206)

Clinical variables	Categories	Frequency (n = 206)	Percent (%)
Asthma control status	Controlled	61	29.6
	Uncontrolled	145	70.4
Smoking status	Yes	13	6.3
	No	193	93.7
Other co morbidities	No	178	86.4
	Hypertension	24	11.7
	DM	4	1.9
Alcohol intake status	Yes	45	21.8
	No	161	78.2
Type of MDI use	Salbutamol	49	23.8
	Beclomethasone	8	3.9
	Both	149	72.3
Duration of MDI use in years	≤ 1	62	30.1
	1–10	100	48.5
	11–25	44	21.4
Frequency of becomethasone	2 times/day	175	85.0
	When worsening	8	3.9
	Never	23	11.2
Health education on MDI use	Yes	179	86.9
	No	27	13.1
Health education given by	Physician	146	70.9
	Pharmacist	17	8.3
	Nurse	43	20.9
Family history of bronchial Asthma	Yes	78	37.9
	No	128	62.1
MDI technique status	Proper	59	28.6
	Improper	147	71.4
Duration of asthma recoded (years)	≤ 1	18	8.7
	1–10	95	46.1
	≥ 10	93	45.1

#### Factors associated with poor MDI techniques

After the bivariate logistic regression analysis (Additional file 3: Table S1 and Additional file 4: Table S2) was multiple logistic regression analysis was then conducted to assess the factors associated with poor MDI technique lack of health education on MDI technique and uncontrolled Asthma status remained significant predictors of poor technique. As it can be seen from Table 3, patients with lack of health education about device technique were about 5 times more likely to have poor technique as compared to those who get the education [95% CI 1.08–22.89]. The other group of patients are those with uncontrolled Asthma who are 3.7 times more likely to have poor technique as compared to people with controlled Asthma [95% CI 1.85–7.23] (Table 3).

**Table 3:** Multivariate analysis of factors associated with MDI techniques among patients with Asthma (n = 206)

Variable	MDI technique		COR (CI: p value)	AOR (CI: p value)	p-value
	Proper, n (%)	Improper, n (%)			
Marital status					
Single	6 (25)	18 (75)	2.43 (0.75–7.92)	2.29 (0.63–8.3)	0.21
Married	33 (25.4)	97 (74.6)	2.39 (1.04–5.49)	1.9 (0.79–4.66)	0.15
Divorced	7 (30.4)	16 (69.6)	1.86 (0.59–5.87)	1.89 (0.55–6.48)	0.31
Widowed	13 (44.8)	16 (55.2)	1		
Health education on MDI use					
Yes	57 (31.8)	122 (68.2)	1		
No	2 (7.4)	25 (92.6)	5.84 (1.34–25.5)	4.96 (1.08–22.89)	*0.04*
Asthma control status					
Controlled	30 (49.2)	31 (50.8)	1		
Uncontrolled	29 (20)	116 (80)	3.87 (2.03–7.39)	3.67 (1.85–7.23)	*0.001*

*AOR *adjusted odds ratio, *CI *confidence interval, *COR *crude odds ratio, *1* referent

### Discussion

Although MDI drugs are the mainstay of treatment in bronchial Asthma, their adequate benefit largely depends on the appropriate technique of administration, and patients tend to use them improperly for different reasons. In the present study, high prevalence of inappropriate device utilization was found among patients with Asthma. This improper practice was significantly associated with lack of health education about the device use steps and poor symptomatic control of bronchial Asthma.

Problems with the inhaler technique had been repeatedly mentioned in relation to poor Asthma control. Overall, in this study, the improper technique was found to be 71.4%. Lack of health education on MDI technique and poor Asthma control was significantly associated with the high prevalence of inappropriate device technique. Despite this high level of poor technique, results from the Indian study were even worse showing device misuse of 94.3% [11]. The difference between these studies was, the later had involved patients with COPD in addition to bronchial Asthma which could influence the result in a negative way because patients with COPD tend to have a less marked response to inhalers than bronchial Asthma.

With regards to the type of medications, around 72.3% of the participants in our study were using both controllers (beclomethasone) and reliever (salbutamol) MDI medications, which was different from the result of study in France, in which majority were taking SABA only [12], this could possibly be explained by the severity of the illness where in our case they tend to come when the disease is severe enough to mandate initiation of controllers and the fact that the later study was done in primary health care setup.

On the other hand the result of this study was similar to the facility based study in France where MDI was misused by 71% of the participants and level of uncontrolled Asthma was also similar (70.4%), but Similar study in Saudi Arabia revealed significantly lower rate of inappropriate technique (45%) as compared to our study and this problem was associated with poor control of Asthma [7, 13], which could be explained by difference in setup and socioeconomic status of the facility and participants. Technique was even better with the Brazilian (30.2%) and Korean (23.4%) studies [14, 15]. The very low prevalence of inappropriate practice in this could be because those studies were done in special Asthma and allergy clinics with routine health education about the disease and medications.

Majority were using the beclomethasone in twice daily basis (85%) and responded as if they were given education on MDI technique (71.4%) despite the poor Asthma control status of 70.4%, which could be because of problems with differentiating proper health education from just simple information during prescription. The high degree of uncontrolled Asthma status in this study is similar to other two Asthma control studies in Jimma (Ethiopia), where 64.5% and 76.1% had uncontrolled Asthma [10, 16]. Improper Asthma MDI technique is common in the study area. Efforts should be done to improve the appropriate Asthma control technique including proper health education on MDI technique should be given to all patients with Asthma and checked routinely at every visit. The hospital administration should assign trained health educators on the proper use of inhalational drug administration which is vital step in Asthma control.

## Limitations

This study was conducted within single public hospital on limited numbers of patients who were at chest clinic follow up which means the findings may not be generalizable to the overall Ethiopian patients attending chest clinics in this or other public or private clinics.

## Additional files

**Additional file 1: Appendix 1.** Operational definitions.

**Additional file 2: Appendix 2.** Data collection tools.

**Additional file 3: Table S1.** Bivariate analysis of factors associated with MDI techniques among patients with Asthma at University of Gondar hospital North West Ethiopia, 2017 (n = 206).

**Additional file 4: Table S2.** Association of MDI technique with Asthma control status among patients Asthma at University of Gondar hospital North West Ethiopia, 2017 (n = 206).

## Abbreviations

ACT: asthma control test
GINA: global initiative for asthma
BMI: body mass index
FEV1: forced expiratory volume in one second
FVC: forced vital capacity
ICU: Intensive Care Unit
IDAT: inhalational device assessment tool
SABA: short acting beta agonist
UOGH: University of Gondar Hospital
WHO: World Health Organization
