# Supplementary material for: Status of metered dose inhaler technique among patients with asthma and its effect on asthma control in Northwest Ethiopia
Source: BMC Res Notes. 2019 Jan 14;12:15. doi: 10.1186/s13104-019-4059-9 (PMC6332522; doi:10.1186/s13104-019-4059-9)
Supplement: Supplementary file 1 — Additional file 1: Appendix 1. Operational definitions. [file 13104_2019_4059_MOESM1_ESM.docx]

**Operational definitions**

. Control of asthma according to Asthma control technique (ACT)

- Well controlled asthma-ACT score of ≥ 20.
- Uncontrolled asthma-ACT score of ≤ 19.

Inhalational Device Assessment Tool (IDAT) Summary each of the five steps for all forms of the IDAT is scored as 1 or 0.

- A step is scored as ‘1’ if no errors are made for that step.
- A step is scored as ‘0’ if there is at least one error in performing that step.
- The scores for the five steps are then added, for a total possible maximum score of 5, and a minimum score of 0.
- Target proper techniques for IDAT- a score of 95% to 100% should be obtained.
