# Supplementary material for: Status of metered dose inhaler technique among patients with asthma and its effect on asthma control in Northwest Ethiopia
Source: BMC Res Notes. 2019 Jan 14;12:15. doi: 10.1186/s13104-019-4059-9 (PMC6332522; doi:10.1186/s13104-019-4059-9)
Supplement: Supplementary file 2 — Additional file 2: Appendix 2. Data collection tools. [file 13104_2019_4059_MOESM2_ESM.docx]

**Questionnaire for studying status of Metered Dose Inhaler (MDI) technique of asthmatic patients and its effect on asthma control in Northwest Ethiopia.**

**Part I. Socio-demographic data**

**1**. Chart number ___________, Chest clinic number ___________

2. Age in years_____

3. Gender: 1. Male 2. Female

5. Religion: 1. Orthodox 2. Muslim 3. Protestant 4. Others____

6. District of residence: 1. Urban 2. Rural

7. Marital status: 1. Single 2.Married 3. Divorced 4. widowed

8. Educational level: 1. No formal education 2. Primary school 3. Secondary school 4. Higher education

9. Occupation: 1. Merchant 2. Civil servant 3. House wife (for female) 4. Student 5. Farmer 6. Not working 7. Daily labor 8. Others

10. Smoking: 1. Yes 2. No

11. Alcohol intake: 1. Yes 2. No

12. Annual income ___________

**Part II. Disease characteristics**

13. Duration since asthma diagnosis in months_____

14. Other documented co-morbidity (Specify)_______

15. History of MDI use? 1. Yes 2. No

16. Type of MDI used? 1. Salbutamol 2. Beclomethasone 3. Both 4. Others (Specify)_______

17. Duration of MDI device use in months____

18. How often do you take your beclomethasone?

1.Two times/day 2. When worsening 3. Never

19. If not two times/day to no. 18 why?

1. Financial problem 2. Lack of knowledge 3. Not available all the time

20. Were you given health education on MDI use ? 1. Yes 2. No

21. If yes, who gave you the health education? 1. Physician 2. Pharmacist 3. Nurse 4. Others

22. If yes to no 20, are you adherent to the exact instruction you were given? 1. Yes 2. No

23. If no to number 22, why? ______

24. Family history of Bronchial Asthma? 1. Yes 2. No

25. Family history of atopy? 1. Rhinitis 2. dermatitis 3. others(Specify_____ ) 4. No atopy

26. Personal history of atopy? 1. Rhinitis 2. Dermatitis 3. Others(Specify_____ ) 4.No atopy

**Part III. Asthma control tecknique parameters**

1. How much of the time did your asthma keep you from getting as much done at work in the past 4 weeks?

1. All of the time 2. Most of the time 3. Some of the time 4. Alittle of the time 5. none

2. How often have you had shortness of breath In the past 4 weeks?

1. > Once/day 2. Once/day 3. 3-6/ week 4. Once or twice/week 5. Not at all

3. How often did your asthma symptoms awaken you at night/morning in the past 4 weeks?

1. ≥ 4 nights/week 2. 2 or 3/week 3. Once/week 4. Once or twice 5. Not at all

4. How often have you used your rescue inhaler medication in the past 4 weeks?

1. ≥ 3x/day 2. 1 or 2x/day 3. 2 or 3x/week 4. ≤ Once/week 5. Not at all

5. How would you rate your asthma control during the past 4 weeks?

1. Uncontrolled

2. Poorly controlled

3. Somewhat controlled

4. Well controlled

5. Completely controlled

**Part IV. IDAT/observe the patient while using the inhaler drug/**

| **Criteria** | **yes=1** | **No=0** |
| --- | --- | --- |
| 1. Removes cap from the mouthpiece |  |  |
| 2. Shakes the inhaler |  |  |
| 3. Exhales completely/breathes out to the end |  |  |
| 4. Inhales appropriately-release 1 puff and Continues breathing in slowly for about 5 seconds |  |  |
| 5. Holds breath for 10 seconds with closed lips |  |  |
