# Supplementary material for: Status of metered dose inhaler technique among patients with asthma and its effect on asthma control in Northwest Ethiopia
Source: BMC Res Notes. 2019 Jan 14;12:15. doi: 10.1186/s13104-019-4059-9 (PMC6332522; doi:10.1186/s13104-019-4059-9)
Supplement: Supplementary file 3 — Additional file 3: Table S1. Bivariate analysis of factors associated with MDI techniques among patients with Asthma at University of Gondar hospital North West Ethiopia, 2017 (n = 206). [file 13104_2019_4059_MOESM3_ESM.docx]

**Table S1: Bivariate analysis of factors associated with MDI techniques among patients with Asthma at University of Gondar hospital North West Ethiopia, 2017(n=206).**

| Variable | | MDI technique | | COR (CI) | P value |
| --- | --- | --- | --- | --- | --- |
|  | | Proper, n (%) | Improper, n (%) |  |  |
| Gender | Males | 22(25.9) | 63(74.1) | 1.26 (0.6-2.3) | 0.4 |
|  | Females | 37(30.6) | 84(69.4) | 1 |  |
| Age | 18-34 | 8 (22.9) | 27(77.1) | 1.36(0.56-3.3) | 0.5 |
|  | 35-54 | 33(28.7) | 82(71.3) | 0.85(0.43-1.70) | 0.6 |
|  | >55 | 18(32.1) | 38(67.9) | 1 |  |
| Religion | Orthodox | 41(25.3) | 121(74.7) | 2.58(0.88-7.56) | 0.08 |
|  | Muslim | 11(37.9) | 18(62.1) | 1.43(0.40-5.06) | 0.58 |
|  | Protestant | 7(46.7) | 8(53.3) | 1 |  |
| Residence | Urban | 45(29.4) | 180(70.6) | 0.86(0.43-1.74) | 0.68 |
|  | Rural | 14(26.4) | 39(73.6) | 1 |  |
| Marital status | Single | 6(25) | 18(75) | 2.43(0.75-7.92) | 0.14 |
|  | **Married** | **33(25.4)** | **97(74.6)** | **2.39(1.04-5.49)** | **0.04** |
|  | Divorced | 7(30.4) | 16(69.6) | 1.86(0.59-5.87) | 0.29 |
|  | Widowed | 13(44.8) | 16(55.2) | 1 |  |
| Educational status | No formal education | 35(31.3) | 77(68.8) | 1.58(0.70-3.57) | 0.27 |
|  | Primary school | 13(26) | 37(74) | 2.22(1.01-4.91) | 0.05 |
|  | Secondary school | 8(21.1) | 30(78.9) | 1.18(0.41-3.41) | 0.77 |
|  | Higher education | 3(50) | 3(50) | 1 |  |
| Occupational | Merchant | 4(16.7) | 20(83.3) | 1.71(0.49-5.86) | 0.39 |
|  | Civil Servant | 14(25.5) | 41(74.5) | 1 |  |
|  | House Wife | 25(32.9) | 51(67.1) | 0.69(0.32-1.51) | 0.36 |
|  | Student | 16(31.4) | 35(68.6) | 0.75(0.32-1.74) | 0.50 |
| Monthly income | <1200 | 27(31) | 60(69) | 0.67(0.17-2.62) | 0.56 |
|  | 1201-2499 | 29(27.4) | 77(72.6) | 0.79(0.21-3.10) | 0.74 |
|  | >=2500 | 3(23.1) | 10(76.9) | 1 |  |
| Smoking status | Yes | 3(23.1) | 10(76.9) | 1.36(0.36-5.14) | 0.65 |
|  | No | 56(29) | 137(71) | 1 |  |
| Alcohol intake | Yes | 13(28.9) | 32(71.1) | 0.99(0.47-2.04) | 0.97 |
|  | No | 46(28.6) | 115(71.4) | 1 |  |
| Other co morbidity | No | 53(29.8) | 125(70.2) | 1 |  |
|  | Hypertension | 5(20.8) | 19(79.2) | 1.61(0.57-4.54) | 0.37 |
|  | DM | 1(25) | 3(75) | 1.27(0.13-12.51) | 0.84 |
| Types of MDI use | Salbutamol | 12(24.5) | 37(75.5) | 1 |  |
|  | Beclomethasone | 1(12.5) | 7(87.5) | 2.27(0.25-20.36) | 0.46 |
|  | Both | 46(30.9) | 103(69.1) | 0.73(0.35-1.52) | 0.39 |
| Duration of MDI device use | <1 year | 20(32.3) | 42(67.7) | 0.75(0.31-1.78) | 0.51 |
|  | 1-10 | 27(27) | 73(73) | 0.96(0.42-2.17) | 0.92 |
|  | 11-25 | 12(27.3) | 32(72.7) | 1 |  |
| Frequency of beclome thasone | 2 times/day | 49(28) | 126(72) | 1 |  |
|  | when worsening | 2(25) | 6(75) | 1.17(0.23-5.97) | 0.85 |
|  | Never | 8(34.8) | 15(65.2) | 0.73(0.29-1.83) | 0.50 |
| Health education on MDI use | Yes | 57(31.8) | 122(68.2) | 1 |  |
|  | No | 2(7.4) | 25(92.6) | **5.84(1.34-25.5)** | **0.02** |
| Health education was given by | Physician | 39(26.7) | 107(73.3) | 1 |  |
|  | Pharmacist | 7(41.2) | 10(58.8) | 0.52(0.18-1.46) | 0.22 |
|  | Nurse | 13(30.2) | 30(69.8) | 0.84(0.39-1.77) | 0.65 |
| Asthma control status | Controlled | 30(49.2) | 31(50.8) | 1 |  |
|  | **Uncontrolled** | 29(20) | 116(80) | **3.87(2.03-7.39)** | **0.001** |
| Duration of Asthma recoded | <1year | 7(38.9) | 11(61.1) | 0.55(0.19-1.57) | 0.26 |
|  | 1-10year | 28(29.5) | 67(70.5) | 0.83(0.44-1.58) | 0.57 |
|  | >10 year | 24(25.8) | 69(74.2) | 1 |  |
