# Supplementary material for: Status of metered dose inhaler technique among patients with asthma and its effect on asthma control in Northwest Ethiopia
Source: BMC Res Notes. 2019 Jan 14;12:15. doi: 10.1186/s13104-019-4059-9 (PMC6332522; doi:10.1186/s13104-019-4059-9)
Supplement: Supplementary file 4 — Additional file 4: Table S2. Association of MDI technique with Asthma control status among patients Asthma at University of Gondar hospital North West Ethiopia, 2017 (n = 206). [file 13104_2019_4059_MOESM4_ESM.docx]

Table S2: Association of MDI technique with Asthma control status

| Variable | Asthma control status | | OR (CI) | P-value |
| --- | --- | --- | --- | --- |
| MDI technique | Controlled | Uncontrolled |  |  |
| Proper | 30 (49.2%) | 29 (20%) | 1 |  |
| Improper | 31 (50.8%) | 116 (80%) | 3.87 (2.03-7.39) | **<0.01** |

Key: CI=Confidence Interval; OR= Odds Ratio; 1= referent
